# Dual role of nNOS in ischemic injury and preconditioning

**DOI:** 10.1186/1472-6793-10-15

**Published:** 2010-08-13

**Authors:** Anupama Barua, Nicholas B Standen, Manuel Galiñanes

**Affiliations:** 1 Cardiac Surgery Unit, Dept. of Cardiovascular Sciences, University of Leicester, UK; 2 Dept. Cell Physiology & Pharmacology, University of Leicester, UK; 3 Department of Cardiac Surgery, Area del Cor (ACOR) and Research Institute, University Hospital Vall d'Hebron, Universitat Autònoma de Barcelona, Barcelona, Spain

## Abstract

**Background:**

Nitric oxide (NO) is cardioprotective and a mediator of ischemic preconditioning (IP). Endothelial nitric oxide synthase (eNOS) is protective against myocardial ischemic injury and a component of IP but the role and location of neuronal nitric oxide synthase (nNOS) remains unclear. Therefore, the aims of these studies were to: (i) investigate the role of nNOS in ischemia/reoxygenation-induced injury and IP, (ii) determine whether its effect is species-dependent, and (iii) elucidate the relationship of nNOS with mitoK_ATP _channels and p38MAPK, two key components of IP transduction pathway.

**Results:**

Ventricular myocardial slices from rats and wild and nNOS knockout mice, and right atrial myocardial slices from human were subjected to 90 min ischemia and 120 min reoxygenation (37°C). Specimens were randomized to receive various treatments (n = 6/group). Both the provision of exogenous NO and the inhibition of endogenous NO production significantly reduced tissue injury (creatine kinase release, cell necrosis and apoptosis), an effect that was species-independent. The cardioprotection seen with nNOS inhibition was as potent as that of IP, however, in nNOS knockout mice the cardioprotective effect of non-selective NOS (L-NAME) and selective nNOS inhibition and also that of IP was blocked while the benefit of exogenous NO remained intact. Additional studies revealed that the cardioprotection afforded by exogenous NO and by inhibition of nNOS were unaffected by the mitoK_ATP _channel blocker 5-HD, although it was abrogated by p38MAPK blocker SB203580.

**Conclusions:**

nNOS plays a dual role in ischemia/reoxygenation in that its presence is necessary to afford cardioprotection by IP and its inhibition reduces myocardial ischemic injury. The role of nNOS is species-independent and exerted downstream of the mitoK_ATP _channels and upstream of p38MAPK.

## Background

It is well established that enhanced bioavailability of endogenous nitric oxide (NO) affords cardioprotection against ischemia and reoxygenation-induced injury [1]. This important mediator is enzymatically produced by the NO-synthases (NOSs). There are three NOS isoforms, widely distributed through most cells and tissues, which can produce NO by converting L-arginine to L-citruline in the presence of NADPH, O_2 _and other co-factors [2]. The endothelial NOS (eNOS) and neuronal NOS (nNOS) are constitutive and Ca++-dependent whereas the expression of the inducible NOS (iNOS) is Ca++-independent and stress-induced.

It has been documented that nNOS is located in the peripheral vagal nerves, sympathetic nerves and in the autonomic control region of the central nervous system [2]. In cardiomyocytes, nNOS is expressed in sarcoplasmic reticulum, sarcolemma and mitochondria [3-5]. NO derived from nNOS influences the beat to beat regulation of basal cardiac function, serving negative feedback control of Ca^++ ^fluxes [6,7]. In addition, it facilitates vagal stimulation and inhibits sympathetic stimulation by exerting its effect in the central and peripheral nervous system.

NO derived from eNOS is essential for eliciting early ischemic preconditioning (IP) [8], however, there is still controversy about the role of nNOS. Thus, although nNOS expression is increased in the acute phase of myocardial infarction in both infarcted and non-infarcted tissues in the rat heart [9], it has been reported that in the nNOS knockout mice, there is no change in myocardial infarct size [10,11]. By contrast, it appears that delayed IP (72 hours) is dependent on nNOS in the rabbit [12], whereas the deficit of nNOS reduces cerebral ischemic injury in mice [13].

MitoK_ATP _channels, located in the inner mitochondrial membrane, are central for IP [14]. Few studies have shown a link between mitoK_ATP _and NO in IP. Sasaki *et al *suggested that NO causes the opening of mitoK_ATP _directly and indirectly [15] which in turn induces blunting of the Ca^++ ^overload in mitochondria, thus contributing to the delayed IP. It has also been reported that the opening of the mitoK_ATP _channels by diazoxide induces cardioprotection in a NO-dependent manner [16]. Furthermore, Rakhit *et al *have demonstrated that the exogenous NO donor SNAP induces early IP by a cGMP-dependent mechanism and delayed IP by a cGMP-independent mechanism which is mediated by activation of PKC and mitoK_ATP _channels [17]. Interestingly, Nakano *et al *suggested that SNAP-induced cardioprotection cannot be reproduced by endogenous NO [18] also implying that there are two separate pathways for NO-induced cardioprotection.

Our laboratory has previously demonstrated that opening of p38MAPK is an obligatory step in IP that lies downstream of PKC activation [19]. However, there is little information regarding the relationship of NO with p38MAPK in the myocardium. Thus, for example, Kim *et al. *showed that sodium nitroprusside activates p42/44 and p38MAPK in adult rat cardiomyocytes via both cGMP-dependent and cGMP-independent mechanisms [20]. In another study, Wang et al demonstrated that NO suppressed the angiotensin-I induced activation of ERK in cardiac fibroblast [21]. Other studies carried out in non-cardiac mitogenic cells have shown that NO induced apoptosis is mediated directly by activation of MAPK [20-22]. So, the relationship of NO with p38MAPK remains largely undefined.

Therefore, the aims of the present studies were to: (i) elucidate the role of nNOS in ischemia/reoxygenation-induced injury and IP, (ii) determine whether its effect is species-dependent, and (iii) investigate the relationship of nNOS with mitoK_ATP _channels and p38MAPK, two key components of the intracellular transduction pathway of protection by IP.

## Results

Myocardial viability, as assessed by the absence of necrosis and apoptosis by the end of the experimental period in aerobically incubated myocardial slices, was greater than 95% in all instances.

### (i) The role of nNOS in ischemic injury

**Figure 1:**
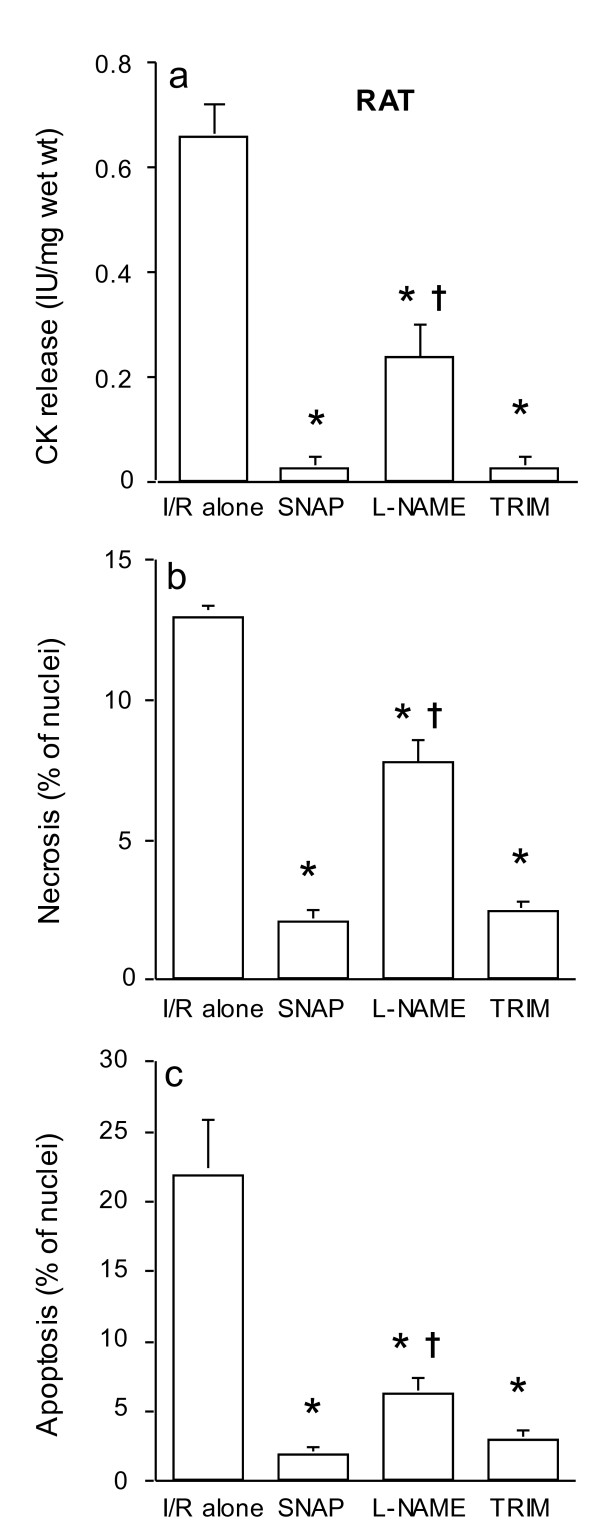
**CK release (a), and cell necrosis (b) and apoptosis (c) in rat ventricular myocardium subjected to 90 min ischemia followed by 120 min of reoxygenation (n = 6/group)**. Specimens were incubated with SNAP, L-NAME or TRIM for 20 min prior to ischemia. *P < 0.05 vs. ischemia/reoxygenation (I/R) alone; †P < 0.05 vs. SNAP and TRIM treated groups.

Figures 1a-c show that, in the rat myocardium, the selective inhibition of nNOS by TRIM resulted in a significant reduction in CK release and in cell necrosis and apoptosis, as compared to the mean values in the ischemia/reoxygenation alone group. The degree of this protection was similar to administration of exogenous NO but greater than the non-selective NOS inhibitor L-NAME.

### (ii) Is the role of nNOS species-dependent?

**Figure 2:**
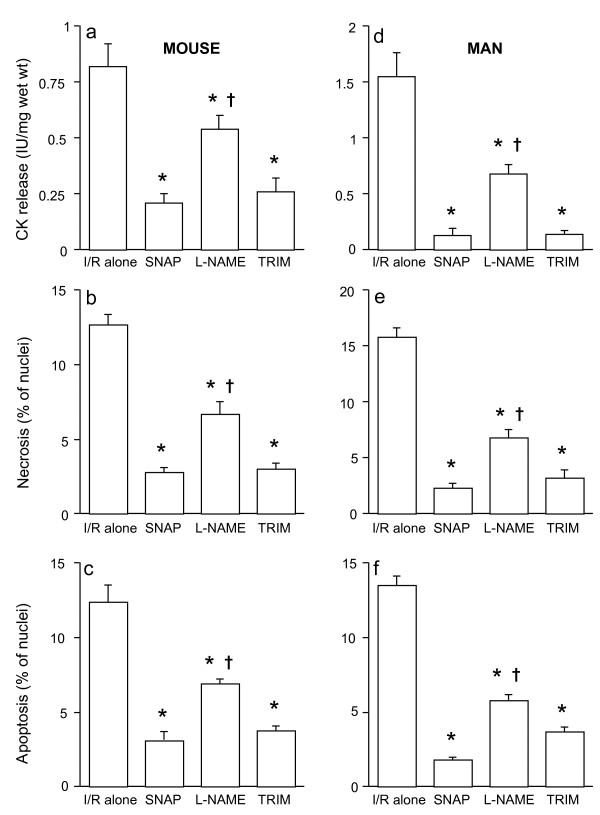
**CK release (a), and cell necrosis (b) and apoptosis (c) in mouse ventricular myocardium and CK release (d), and cell necrosis (e) and apoptosis (f) in human right atrial myocardium subjected to 90 min ischemia followed by 120 min of reoxygenation (n = 6/group)**. Specimens were incubated with SNAP, L-NAME or TRIM for 20 min prior to ischemia. *P < 0.05 vs. ischemia/reoxygenation (I/R) alone; †P < 0.05 vs. SNAP and TRIM treated groups.

As seen in Figures 2a-f, and in comparison with the results observed in Figures 1a-c, the order of magnitude of ischemic injury for CK release and cell necrosis and apoptosis varied slightly between species, however, identical effects on NOS inhibition and from the administration of exogenous NO to that seen in rat myocardium (Figures 1a-c) were observed for both mouse and human myocardium.

### (iii) Knocking out nNOS

**Figure 3:**
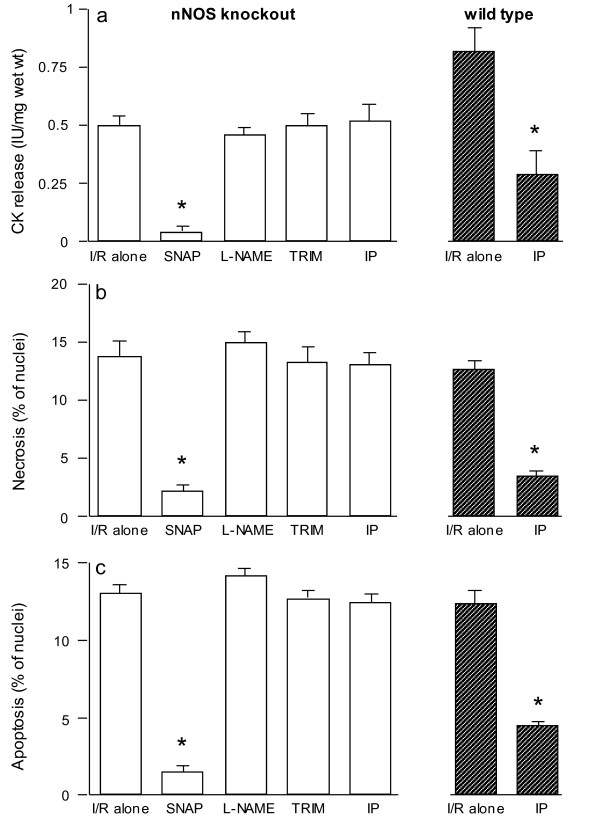
**CK release (a), and cell necrosis (b) and apoptosis (c) in nNOS knockout mice ventricular myocardium subjected to 90 min ischemia followed by 120 min of reoxygenation (n = 6/group)**. Specimens were incubated with SNAP, L-NAME or TRIM for 20 min prior to ischemia. For comparison, some muscles were subjected to ischemic preconditioning (IP). *P < 0.05 vs. ischemia/reoxygenation (I/R) alone.

Figures 3a-c demonstrate that, as opposed to wild type mice, the myocardium of nNOS knockout mice could not be protected by the specific NOS inhibitor TRIM as the mean values for CK release and cell necrosis and apoptosis were not significantly different from muscles subjected to ischemia/reoxygenation alone. The absence of benefit when L-NAME was added to the myocardium of knockout nNOS mice supports the thesis that although the endogenously produced NO by eNOS may be beneficial its deficit did not cause further ischemic injury. As seen above, again the NO donor SNAP almost abolished the CK release and the cell death induced by ischemia and reoxygenation. Importantly, and in contrast with wild type mice, the myocardium of nNOS knockout out mice could not be protected by IP since the mean values for CK release and cell necrosis and apoptosis did not differ from the values in the ischemia/reoxygenation alone group. However, the degree of injury in the ischemia-reoxygenation alone groups was similar in both wild type and nNOS knockout mice as previously reported [10].

### (iv) The relationship of exogenous NO and nNOS inhibition with mitoK_ATP _channels and p38MAPK

**Figure 4:**
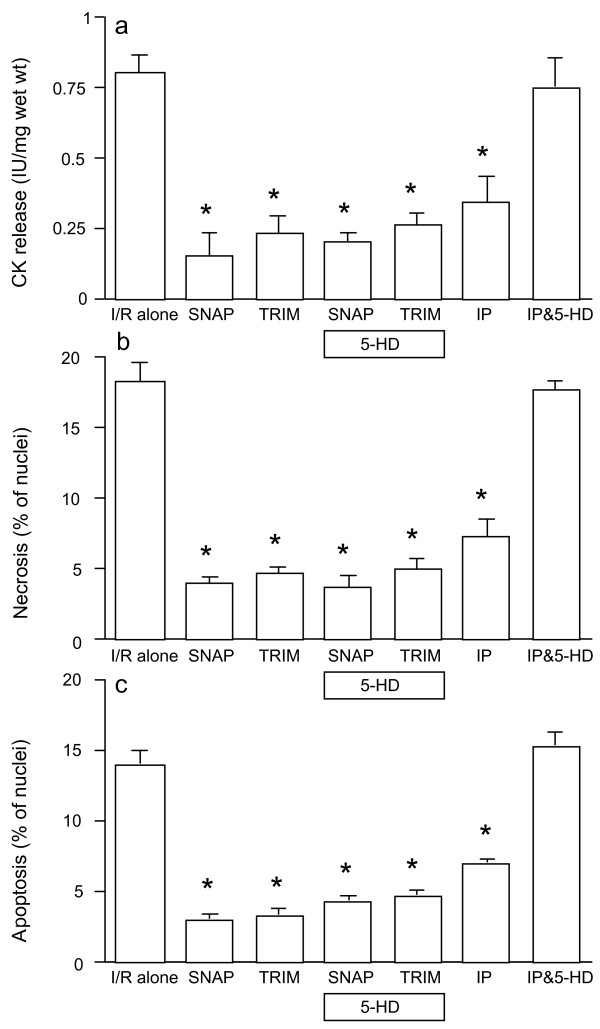
**CK release (a), and cell necrosis (b) and apoptosis (c) in rat ventricular myocardium subjected to 90 min ischemia followed by 120 min of reoxygenation (n = 6/group)**. Specimens were incubated with SNAP or TRIM for 20 min prior to ischemia in the absences and presence of 5-HD. For comparisons, some muscles were subjected to ischemic preconditioning (IP) in the absences and presence of 5-HD. *P < 0.05 vs. ischemia/reoxygenation (I/R) alone.

**Figure 5:**
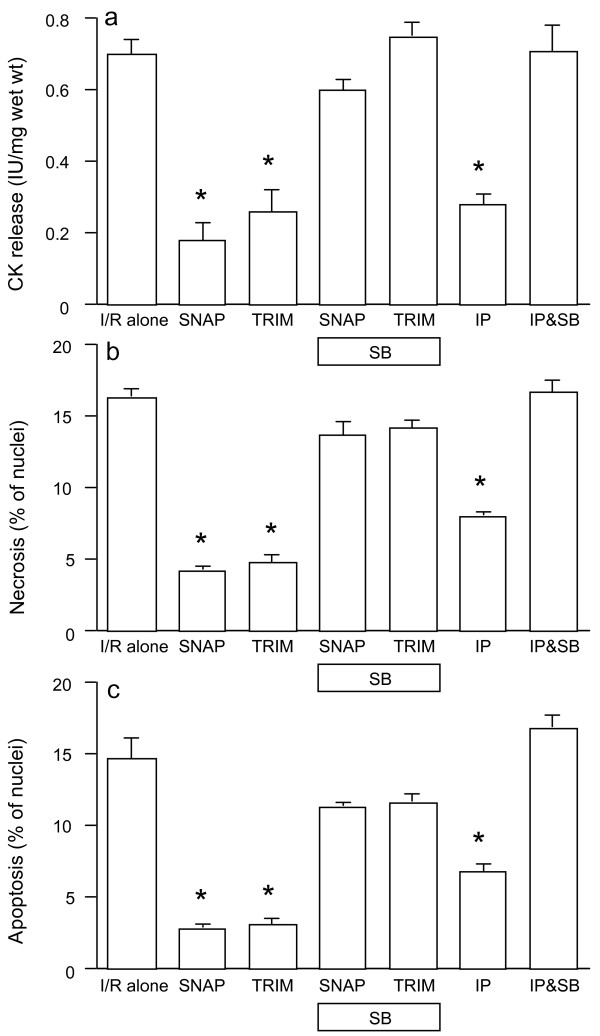
**CK release (a), and cell necrosis (b) and apoptosis (c) in rat ventricular myocardium subjected to 90 min ischemia followed by 120 min of reoxygenation (n = 6/group)**. Specimens were incubated with SNAP or TRIM for 20 min prior to ischemia in the absences and presence of SB203580 (SB). For comparisons, some muscles were subjected to ischemic preconditioning (IP) in the absences and presence of SB203580. *P < 0.05 vs. ischemia/reoxygenation (I/R) alone.

Figures 4a-c demonstrate that the reduction in CK release and in cell necrosis and apoptosis caused by the NO donor SNAP and the inhibition of nNOS with TRIM, was unaffected by the mitoK_ATP _channel blocker 5-HD, and that, as expected, it blocked the protection by IP. By contrast, Figures 5a-c show that the p38MAPK blocker SB203580 abolished the protective effect of exogenous NO and of nNOS inhibition, along with the benefit of IP.

## Discussion and Conclusion

The present studies have demonstrated that nNOS plays a dual role so that its presence is necessary to afford cardioprotection by IP, but at the same time its inhibition reduces myocardial ischemic/reoxygenation induced injury. In addition, they have shown that the protection obtained by the inhibition of nNOS is as potent as the exogenous administration of NO and that of IP, effects that are species-independent. Importantly, the action of nNOS and those of exogenous NO are exerted downstream of mitoK_ATP _channels and upstream of p38 MAPK.

The duplicity of nNOS-derived effects seen in our studies can be explained, on one hand by a physiological production of NO that triggers the cardioprotective intracellular mechanism of interventions such as IP, and on the other hand by a possible much greater production of NO induced by ischemia/reoxygenation that is detrimental. In this connection, other investigators have also reported in an infarct size model that nNOS plays an opposite role in ischemia/reperfusion and IP [23] and that the protection of delayed IP in the rabbit heart is dependent on nNOS [12]. Since NO donors such as SNAP also have strong cardioprotective effects, it may be speculated that the amount and cell location of NO production would determine whether the final result is tissue protection or injury. It is also interesting to note that myocardial injury (CK release and cell death) was not increased by ischemia/reoxygenation in nNOS knockout mice, a finding supported by the absence of changes in infarct size in nNOS knockout mice [9,11]. By contrast, a deficit in nNOS in mice reduced the degree of cerebral ischemic injury [13], thus suggesting that the role of nNOS in ischemia/reoxygenation could be organ-specific.

Our studies locate the action of nNOS downstream of the mitoK_ATP _channels since the protection induced by nNOS specific inhibitor TRIM was blocked by 5-HD, a mitoK_ATP _channel inhibitor. The finding that exogenous NO is also acting at the same point reinforces the view that the production of endogenous NO by nNOS is the mechanism of protection. Indeed, other laboratories have shown that the protection induced by exogenous NO is not affected by blockade of mitoK_ATP _channels with 5HD or glibenclamide [17] and that the protection induced by the mitoK_ATP _channel opener diazoxide is NO-dependent [14], further supporting the thesis that NO metabolism is a critical component of the cell survival mechanism. The different location of the two constitutive forms of NOS, nNOS and eNOS, may also suggest that NO plays a distinctive role depending on the cellular site where it is produced. This thesis gets support from the finding that the specific inhibition of nNOS was more protective than the non-specific inhibition of NOS. Certainly, this is an important question that would require further investigations.

Previously, our laboratory has shown that p38MAPK is another critical factor of IP-induced cardioprotection located beyond the mitoK_ATP _channels [19,24]. Here we have also shown that the effect of NO metabolism in the intracellular signaling pathways of cardioprotection is exerted upstream of p38MAPK, as the beneficial effects of exogenous NO donation and nNOS inhibition were abolished by the p38MAPK blocker SB203580. These findings are supported by the demonstration of protection of cardiac [20] and non-cardiac cells [22,25], by NO through activation of p38MAPK and other MAPKs.

An additional important finding is the identical role played by nNOS in the three species studied (rat, mouse and human), suggesting that the function of the enzyme has been preserved throughout evolution. These results may have clinical implications since the findings observed from the animal studies could be extrapolated to human beings without the need for additional confirmatory investigations. Nonetheless, it is worth emphasizing that the present studies were performed in an *in vitro *model and therefore care must be taken when extrapolating the present results to clinical conditions. However, despite the differences between atrial and ventricular samples, the myocardium from the mouse and rat ventricles and from the human right atrial appendage, responded in a similar manner and hence the different source of myocardium should not represent a problem for the interpretation of the results. During cardiac surgery, myocardial ischemia is induced in a controlled manner and therefore it would be possible to enhance cardioprotection by manipulation of the NO metabolism. However, in the clinical setup, the heart may be subjected to stresses that can result in the expression of the inducible NOS (iNOS) and it would be important to elucidate the role of the constitutive NOS isoforms, nNOS and eNOS, in the pathophysiology of ischemia/reperfusion-induced injury under such conditions. It is worth emphasizing that, although the activity of nNOS has been shown to be increased in the late phase of delayed IP[12], to the best of our knowledge, the activity of the enzyme has not been determined in early IP. Indeed, it would be important to determine the activity of all NOS isoforms in future studies.

In conclusion, here we have shown that nNOS is a critical factor playing a dual role by participating in myocardial ischemia/reoxygenation injury, and at the same time, by promoting myocardial protection. These results are of clinical relevance for the design of therapeutic interventions aimed at reducing the myocardial damage caused by ischemia/reoxygenation.

## Methods

### Procurement of myocardial samples

Wister rats and C57BL/6J mice were purchased from Charles Rivers UK Ltd (Kent, UK). nNOS knockout mice (C57BL/6J) were also obtained from The Jackson Lab (Bar Harbor, Maine, USA). Animals were culled by cervical dislocation and the heart was rapidly dissected and sectioned as described below. These studies were undertaken in accordance with the guidelines on the Operation of Animals (Scientific Procedure) Act 1986. All procedures are approved by the Animal Care and Use Committee of the University of Leicester.

The right atrial appendage from patients undergoing elective heart surgery for coronary bypass surgery or aortic valve surgery was obtained prior to the initiation of cardiopulmonary bypass. Patients with atrial fibrillation, cancer, diabetes, poor LV function (EF < 30%) or with additional surgical procedures or those being treated with opioids, catecholamines, or the mitoK_ATP _channel opener nicorandil were excluded from the study. The study was conducted according to Declaration of Helsinki principles and approval was obtained from the Local Ethics Committee. All participants provided informed written consent.

### Experimental model and preparation of myocardial samples

The experimental preparation has been previously described and characterized [26,27]. Briefly, upon harvesting, samples (rats and mice hearts and human right atrial appendages) were immediately immersed in cold (4°C) Krebs/Henseleit/Hepes (KHH) buffered medium containing (in mM): NaCl 118, KCl 4.8, NaHCO_3 _27.2, KH_2_PO_4 _1, MgCl_2 _1, CaCl_2 _1.25, Glucose 10 and Hepes 20, pre-bubbled with 95% O_2_/5% CO_2 _and at a pH of 7.4. Specimens were immediately sectioned manually with a skin-graft blade (Swann-Morton Ltd, Sheffield, U.K.) to slices of 30-50 mg weight and 300-500 μm thickness. The specimens and the apparatus were kept wet during sectioning with cold and oxygenated medium. After this, specimens were equilibrated under normothermic aerobic conditions (95% O_2_/5% CO_2_) for 50-60 min, and then subjected to 90 min of simulated ischemia at 37°C, obtained by continuously bubbling the media with 95% N_2_/5% CO_2 _in the absence of glucose and at a pH of 6.8, followed by 120 min reoxygenation. Time matched aerobic controls were used in every experiment.

For the induction of IP, specimens were subjected to 5 min ischemia followed by 5 min reoxygenation prior to induction of the 90 min ischemia, a protocol that induces maximal protection in this *in vitro *preparation [28]. Reagents were incubated with the specimens prior to ischemia, during ischemia, or during reoxygenation in the manner and at the doses described below in the study protocols.

### Assessment of myocardial injury

Myocardial injury was assessed by measurement of creatine kinase (CK) release into the media during the 120 min reoxygenation period. The enzyme activity was measured by an ultraviolet method based on the formation of NADP employing a commercial assay kit (30-3060/R2: Abbott Laboratories, Diagnostic Division, Kent, UK) and a 96 well flat-bottom micro plate (Costar, Corning Life Sciences, Lowell, Massachusetts, USA). In this assay, NADP is reduced to NADPH and absorbance at 340 nm was measured using a spectrophotometer (Benchmark, Bio-Rad Laboratories, California, USA). Results were expressed as IU/mg wet weight.

### Assessment of cell death

At the end of the experimental protocol, specimens were incubated for 15 min at room temperature on a rocker with 20 μg/ml propidium iodide (PI, Sigma-Aldrich, Dorset, UK) in phosphate buffered saline (PBS) at a pH of 7.4, to identify the necrotic nuclei. Specimens were then washed with PBS twice for 5 min each time before fixation in 4% paraformaldehyde. They were kept overnight at 4-10°C and then transferred to sucrose 30% until the tissue sank. All the above steps were performed in the dark to avoid exposure to light. Following this, the specimens were embedded with Optical Cutting Temperature embedding matrix (Tissue-Tek^®^, Agar Scientific Ltd, Essex, UK). Frozen sections were then cut at 7 μm thickness in a Bright cryotome (model OTF) at circa -25°C, and sections were collected on SuperFrost Plus slides (Menzel Glasser, Braunschweig, Germany). During the preparation of slides, the periphery of specimens were discarded because this area may be more susceptible to damage by the handling. The slides were then kept at -80°C until further analysis.

To assess apoptosis, the slides were brought from -80°C to room temperature, washed with 20 mM PBS, and then permeabilised for 1 minute in a microwave oven at 850 watts in 200 ml of 0.1% Triton X-100 in 0.1 M Tri-sodium citrate buffer at a pH of 6.0. After this, the slides were rapidly cooled by adding 80 ml distilled water and transferred to 20 mM PBS solution. In addition, they were immersed into 3% bovine serum albumin (Sigma Aldrich, Dorset, UK) in 0.1 M Tris-HCl buffer with 20% fetal bovine serum (Hyclone, Utah USA) at a pH of 7.5 for 30 min to block unspecific labeled activity. The terminal deoxynucleotidyl transferase (TdT) was used to incorporate fluorescein (FITC) labelled dUTP oligonucleotides to DNA strand breaks at the 3'-OH termini in a template dependent manner (TUNEL technique) for 90 min at 37°C in a humidity chamber using a commercially available kit (Roche Diagnostics, Penzberg, Germany).

To distinguish the total number of nuclei, sections were counter-stained with 1 μg/ml 4', 6-Diamidino-2-phenylindole (DAPI) (Molecular Probes, Eugene, Oregon, USA) in PBS for 1 min. Then the slides were washed 3 times for 5 min each in PBS. To reduce photobleaching, the sections were mounted with anti-fade solution (Prolong Antifade kit, Molecular Probes, Eugene, Oregon, USA) and covered with coverslips (Menzel Glasser, Braunschweig, Germany).

A fluorescent microscope (Axiovert 200 M, Zeiss fluorescent microscope, Göttingen, Germany) at 40× magnification was used to assess necrosis and apoptosis. At least ten fields per section, and one section per staining, were examined for each experiment. The fields were measured following the horizontal and vertical axes of the sections. PI and FITC labelled nuclei were detected by the Cy3 and EGFP channels respectively, whilst DAPI labelling was detected by the DAPI channel. Only the necrotic or apoptotic signals coinciding with DAPI were considered true events. The NIH Image software (Scion Corp, Frederick, Maryland, USA) was used to determine the total events for each field. To avoid the inclusion of artefacts, only fluorescent signals with areas greater than 16 μm^2 ^were counted.

### Study protocols

In each study, specimens were randomly allocated to different groups (n = 6/group) and specimens from one single donor were not utilized twice for the same group. Some specimens were aerobically incubated for the whole experimental time under aerobic conditions; and others, subjected to 90 min ischemia/120 min reoxygenation alone, served as ischemic controls. The following studies were sequentially carried out:

#### (i) Study 1: The role of nNOS in ischemic injury

To investigate this, specimens from ventricles of rat hearts were randomized to receive the exogenous NO donor SNAP (100 μM), the non-selective NOS inhibitor L-NAME (100 μM), or the selective nNOS inhibitor TRIM (100 μM), for 20 min prior to ischemia. The selected concentrations of the agents used were found to be optimally effective in previous studies [26,28].

#### (ii) Study 2: To elucidate whether the role of nNOS is species-dependent

This was achieved by applying a protocol identical to study 1 to specimens from mice hearts and from human right atrial appendages.

#### (iii) Study 3: To further characterize the role of nNOS using nNOS knockout mice

In this study, nNOS knockout mice were used to further characterize the role of nNOS following a protocol identical to the one in study 1. IP was also applied in a group of specimens. In addition and for comparison, ischemia/reperfusion alone and IP were induced in specimens from wild type mice.

#### (iv) Study 4: To investigate the location of the action of exogenous NO and of nNOS inhibition in relation to mitoKATP channels and p38MAPK

For this study, myocardial slices were obtained from rat ventricles. Specimens were randomized to receive the NO donor SNAP (100 μM), or the selective nNOS inhibitor TRIM (100 μM), for 20 min prior to ischemia in the absence and presence of the mitoK_ATP _channel blocker 5-HD (1 mM) or the p38MAPK inhibitor SB203580 (10 μM) for 30 min. In addition, some preparations were subjected to IP.

### Statistical analysis

Data are expressed as mean ± SEM. Each reported value was obtained after subtracting the corresponding time-matched aerobic control value. One way ANOVA, followed by Bonferroni's test, was used to compare the significance between groups. Analyses were performed using the SPSS program. Differences were considered to be statistically significant if *p *< 0.05.

## Authors' contributions

AB contributed to the design of the study, carried out the laboratory work, collated and analyzed the data and drafted the manuscript. NBS and MG contributed to the design of the study, interpretation and presentation of the data and the writing of the manuscript. All authors have read and approve the final manuscript.
